# Monopolar versus bipolar transurethral resection of bladder tumors: An updated systematic review and meta-analysis of existing studies

**DOI:** 10.1097/MD.0000000000021768

**Published:** 2020-08-21

**Authors:** Jianeng Xu, Junbiao Zheng, Yucheng Ma

**Affiliations:** a Urology Surgery Department, Jiashan First People's Hospital, Jiashan City, Zhejiang Province; b Urology Surgery Department, West China Hospital, Nanchong, Sichuan, China.

**Keywords:** meta-analysis, non-muscle invasive bladder cancer, transurethral resection

## Abstract

**Background:**

To compare the efficacy and safety of bipolar and monopolar transurethral resection of bladder tumors (TURBT) in non-muscle invasive bladder cancer (NMIBC) treatment.

**Methods:**

This protocol established in this study has been reported following the preferred reporting items for systematic review and meta-analysis protocols. Web of Science, PubMed, EMBASE, and the Cochrane Library were searched for all randomized controlled trials comparing bipolar TURBT and monopolar TURBT in NMIBC treatment until 31st of June 2020. We will use a combination of Medical Subject Heading and free-text terms with various synonyms to search based on the eligibility criteria. Two investigators independently reviewed the included studies and extracted relevant data. The odds ratio and 95% confidence intervals of were used as effect estimate. *I*-square (*I*^2^) test, substantial heterogeneity, sensitivity analysis, and publication bias assessment will be performed accordingly. Stata 15.0 and Review Manger 5.3 are used for meta-analysis and systematic review.

**Results:**

The results will be published in a peer-reviewed journal.

**Conclusion:**

The results of this review will be widely disseminated through peer-reviewed publications and conference presentations. This evidence may also provide helpful evidence of the efficacy and safety of bipolar and monopolar transurethral resection of TURBT in NMIBC treatment.

**PROSPERO registration number:**

CRD42020151997

## 1 Introduction

Bladder cancer (BC), one of the most common urological malignancies,^[1]^ is the seventh most frequently encountered for men worldwide. It is the eleventh most common cancer considering both sexes, with an age-standardized mortality rate of 3.2 (per 100,000 person-years) in men and 0.9 in women in 2012.^[2]^ Approximately 75% of patients with BC present with non-muscle invasive bladder cancer (NMIBC), which is comprised of Ta, T1, or carcinoma in situ according to the Tumor, Node, Metastasis classification system.^[3]^ Transurethral resection of the bladder tumor (TURBT) is the gold standard in the diagnosis of NMIBC, and often reported as a crucial treatment for NMIBC.^[4]^ TURBT is conventionally performed with monopolar electrocautery, which requires that the current flows from the resection electrode through the patient's body to the electrode located on the skin. It uses high voltage for tissue cutting and nonconductive and hypotonic fluid for irrigation. However, severe complications resulting from monopolar TURBT (mTURBT), such as TUR syndrome caused by the excessive absorption of fluid into the systemic circulation, a potentially fatal complication in elderly patients with BC (>5 cm), have been increasingly reported.^[5]^ Contrarily, bipolar resection was reported to improve in many aspects, such as better hemostasis, less thermal damage.^[6,7]^ This is because the current flows between 2 electrodes on the resection loop during bipolar TURBT (bTURBT), applying normal saline for irrigation to reduce the incidence of TUR-syndrome,^[8]^ requiring lower power for tissue cutting, and creating a vapor layer in saline to minimize the risk of obturator jerk and further damage.

Despite this, whether bTURBT can completely replace mTURBT as a safer and more effective NMIBC treatment remains controversial.^[9]^ Besides, in previous studies and other meta-analyses comparing mTURBT with bTURBT, the sample size was small, and the source of the research object was also not sufficiently international. Namely, solid evidence to demonstrate the superiority of bTURBT from mTURBT in patients with NMIBC is still lacking. Additionally, medical expenses in BC rank first in those in urological malignancies, which again emphasizes the importance of high-quality TURBT in NMIBC treatment.^[10]^ Therefore, we are committed to the latest systematic reviews and meta-analysis to determine the safer and more effective NMIBC treatment between mTURBT and bTURBT.

## 2 Study aim

The aim of our study is to compare the efficacy and safety of bipolar and mTURBT in NMIBC treatment.

## 3 Methods

The protocol of our meta-analysis followed the guideline of the preferred reporting items for systematic review and meta-analysis protocols recommendations.^[11]^ It has been registered with International Prospective Register of Systematic Reviews (PROSPERO) as CRD42020151997 (https://www.crd.york.ac.uk/prospero/display_record.php?ID=CRD42020151997).

### 3.1 Search strategy

A systematic search was performed in PubMed, Web of Science, Cochrane Library and Embase until 31st of March 2020. The MeSH search and text word will be used with the terms related to “bladder” and “transurethral resection.” To perform a comprehensive and focused search, experienced systematic review researchers will be invited to develop a search strategy. An example of search strategy for PubMed database shown in Table 1 will be modified and used for the other databases. The reference lists of all relevant studies will be searched for additional relevant studies not retrieved from the electronic database search.

**Table 1:**
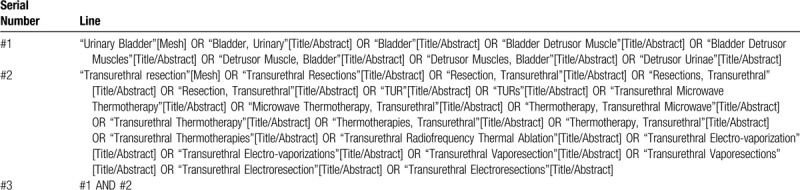
Searching strategy in PubMed.

### 3.2 Eligibility criteria

Inclusion criteria:

(1) it is a randomized controlled trial,
(2) it compares bTURBT with mTURBT,
(3) patients were diagnosed with NMIBC,
(4) at least 1 outcome of interest and related data was reported.

Studies were excluded if:

(1) the patients were diagnosed with muscle-invasive BC,
(2) bTURBT were not compared with mTURBT,
(3) it is a review article, commentaries, and editorials.

### 3.3 Study selection

**Figure 1:**
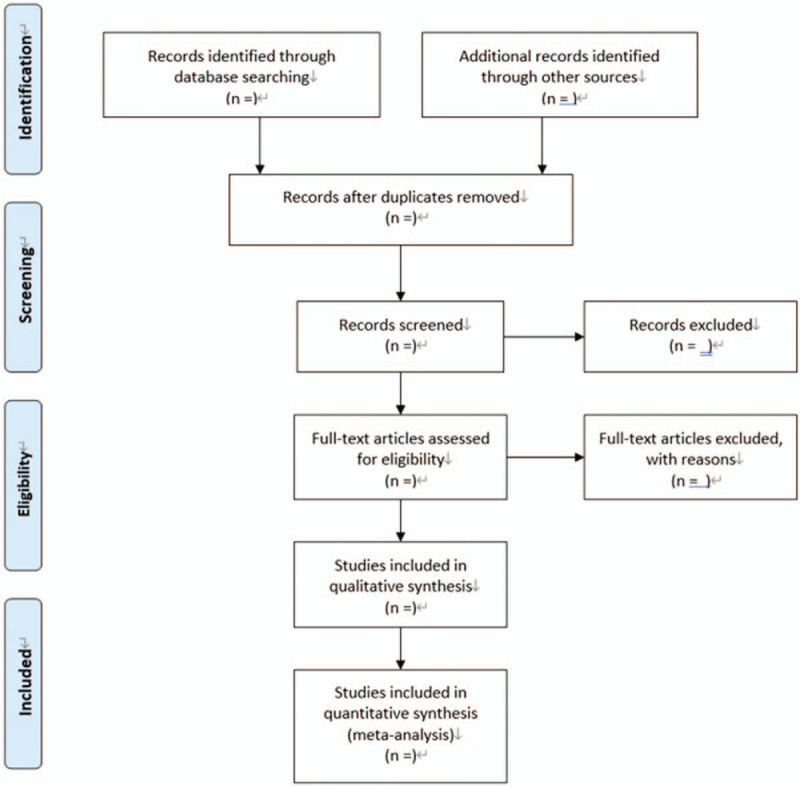
Flow diagram: selection process for the studies.

All initial records from 4 electronic databases will be imported into the web-based systematic review Rayyan software.^[12]^ First, the titles and abstracts of records will be reviewed independently by 2 reviewers to identify potential trials according to eligibility criteria. Then, full-text of all potentially relevant trials will be downloaded to make sure eligible trials. Any conflict will be resolved by discussion. A flow diagram (Fig. 1) will be used to describe the selection process of eligible papers.

### 3.4 Data extraction and management

The data will be extracted out by 2 independent reviewers in accordance with the Cochrane Handbook of Systematic Reviews of Interventions. Two investigators will independently screen all the included studies. We evaluated 2 types of indicators. Operative time, hospitalization time, catheterization time, and recurrence rate are related to effectiveness, while obturator jerk, bladder perforation, thermal damage, and overall complications are connected with safety.

### 3.5 Risk of bias of individual study and quality assessment

Two reviewers will evaluate independently the risk of bias of included studies using a modified version of Cochrane tool^[13]^ in which we will to check for allocation concealment, blinding, incomplete outcome data, selective reporting, and other bias, each of which makes high risk, low-risk, and unclear grades. Any discrepancy was resolved by discussion or by a third reviewer.

### 3.6 Data analyses

All the statistical analysis was achieved in Rev Man 5.3 (Cochrane Library Software, Oxford, UK). The trial data were processed according to the Cochrane Reviewers’ Handbook. We calculated standard deviations based on 95% confidence interval or *P*-values if not reported. Dichotomous data were expressed as odds ratio, whilst continuous variables were presented as mean difference, both with 95% confidence interval. The *z* test was performed to determine all pooled effects, and statistical significance was defined as *P* < .05. If *I*^2^ < 50% or *P* > .1 was reported according to the Chi-square-based *Q* test and *I*^2^ test, heterogeneity was assessed as low, and the fixed-effects model was used. Otherwise, the random-effects model was used. Certain literature was removed each time for sensitivity analysis.

### 3.7 Publication bias

If included studies were more than 10, funnel plot will be used to identify the possible publication bias. Additionally, Egg regression and Begg tests will be utilized to detect the funnel plot asymmetry.^[14]^

### 3.8 Subgroup analysis

If there is enough research, we will conduct a subgroup analysis to investigate differences in age, gender, and so on.

## 4 Discussion

Previous studies have reported that the operative time of bTURBT is significantly shorter than that of mTURBT.^[15]^ This is because of the capacity of rapid hemostasis to provide a clean surgical area. Besides, in bTURBT, adhesion of residual debris to the resectoscope is less likely to occur, and even if it occurs, it is quickly removed without manual removal as slowly as in mTURBT.^[16]^ However, we believe that there is no significant difference in operative time between the 2 groups possibly because bTURBT uses a smaller loop, which requires more time than mTURBT.^[17]^ Moreover, a shorter operative time in the bTURBT group was reported in a meta-analysis, 1 to 2 minutes interval, which is statistically but not clinically significant.^[18]^

Thus, this systematic review and meta-analysis will compare the efficacy and safety of bipolar and mTURBT in NMIBC treatment. The results of this review will be widely disseminated through peer-reviewed publications and conference presentations. This evidence may also provide helpful evidence of whether bTURBT or mTURBT would be regarded as a safer and more effective treatment for patients.

## Author contributions

**Acquisition:** Jianeng Xu, Junbiao Zheng.

**Conceptualization:** Jianeng Xu, Junbiao Zheng, Yucheng Ma.

**Methodology:** Jianeng Xu.

**Project administration:** Jianeng Xu, Junbiao Zheng, Yucheng Ma.

**Registration:** Yucheng Ma.

**Writing & original draft:** Jianeng Xu, Junbiao Zheng.
